# Supplementary figures and images for: KymoButler, a deep learning software for automated kymograph analysis (part 2 of 4)
Source: eLife. 2019 Aug 13;8:e42288. doi: 10.7554/eLife.42288 (PMC6692109; doi:10.7554/eLife.42288)

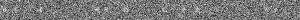

Supplement: Figure 2—source data 2. — A ZIP file containing all analysed synthetic unidirectional movies, their kymographs, results from KymographClear based analysis and manually annotated ImageJ rois. [file elife-42288-fig2-data2.zip › N30/mov07/frame236.tif]

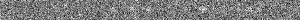

Supplement: Figure 2—source data 2. — A ZIP file containing all analysed synthetic unidirectional movies, their kymographs, results from KymographClear based analysis and manually annotated ImageJ rois. [file elife-42288-fig2-data2.zip › N30/mov07/frame222.tif]

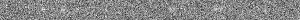

Supplement: Figure 2—source data 2. — A ZIP file containing all analysed synthetic unidirectional movies, their kymographs, results from KymographClear based analysis and manually annotated ImageJ rois. [file elife-42288-fig2-data2.zip › N30/mov07/frame56.tif]

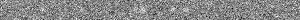

Supplement: Figure 2—source data 2. — A ZIP file containing all analysed synthetic unidirectional movies, their kymographs, results from KymographClear based analysis and manually annotated ImageJ rois. [file elife-42288-fig2-data2.zip › N30/mov07/frame168.tif]

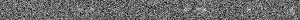

Supplement: Figure 2—source data 2. — A ZIP file containing all analysed synthetic unidirectional movies, their kymographs, results from KymographClear based analysis and manually annotated ImageJ rois. [file elife-42288-fig2-data2.zip › N30/mov07/frame42.tif]

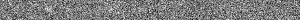

Supplement: Figure 2—source data 2. — A ZIP file containing all analysed synthetic unidirectional movies, their kymographs, results from KymographClear based analysis and manually annotated ImageJ rois. [file elife-42288-fig2-data2.zip › N30/mov07/frame140.tif]

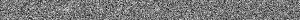

Supplement: Figure 2—source data 2. — A ZIP file containing all analysed synthetic unidirectional movies, their kymographs, results from KymographClear based analysis and manually annotated ImageJ rois. [file elife-42288-fig2-data2.zip › N30/mov07/frame154.tif]

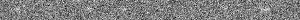

Supplement: Figure 2—source data 2. — A ZIP file containing all analysed synthetic unidirectional movies, their kymographs, results from KymographClear based analysis and manually annotated ImageJ rois. [file elife-42288-fig2-data2.zip › N30/mov07/frame95.tif]

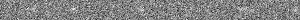

Supplement: Figure 2—source data 2. — A ZIP file containing all analysed synthetic unidirectional movies, their kymographs, results from KymographClear based analysis and manually annotated ImageJ rois. [file elife-42288-fig2-data2.zip › N30/mov07/frame81.tif]

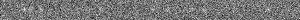

Supplement: Figure 2—source data 2. — A ZIP file containing all analysed synthetic unidirectional movies, their kymographs, results from KymographClear based analysis and manually annotated ImageJ rois. [file elife-42288-fig2-data2.zip › N30/mov07/frame183.tif]

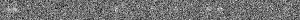

Supplement: Figure 2—source data 2. — A ZIP file containing all analysed synthetic unidirectional movies, their kymographs, results from KymographClear based analysis and manually annotated ImageJ rois. [file elife-42288-fig2-data2.zip › N30/mov07/frame197.tif]

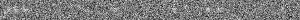

Supplement: Figure 2—source data 2. — A ZIP file containing all analysed synthetic unidirectional movies, their kymographs, results from KymographClear based analysis and manually annotated ImageJ rois. [file elife-42288-fig2-data2.zip › N30/mov07/frame196.tif]

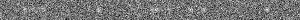

Supplement: Figure 2—source data 2. — A ZIP file containing all analysed synthetic unidirectional movies, their kymographs, results from KymographClear based analysis and manually annotated ImageJ rois. [file elife-42288-fig2-data2.zip › N30/mov07/frame182.tif]

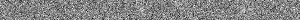

Supplement: Figure 2—source data 2. — A ZIP file containing all analysed synthetic unidirectional movies, their kymographs, results from KymographClear based analysis and manually annotated ImageJ rois. [file elife-42288-fig2-data2.zip › N30/mov07/frame80.tif]

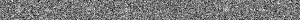

Supplement: Figure 2—source data 2. — A ZIP file containing all analysed synthetic unidirectional movies, their kymographs, results from KymographClear based analysis and manually annotated ImageJ rois. [file elife-42288-fig2-data2.zip › N30/mov07/frame94.tif]

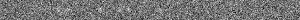

Supplement: Figure 2—source data 2. — A ZIP file containing all analysed synthetic unidirectional movies, their kymographs, results from KymographClear based analysis and manually annotated ImageJ rois. [file elife-42288-fig2-data2.zip › N30/mov07/frame155.tif]

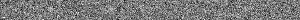

Supplement: Figure 2—source data 2. — A ZIP file containing all analysed synthetic unidirectional movies, their kymographs, results from KymographClear based analysis and manually annotated ImageJ rois. [file elife-42288-fig2-data2.zip › N30/mov07/frame141.tif]

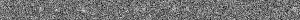

Supplement: Figure 2—source data 2. — A ZIP file containing all analysed synthetic unidirectional movies, their kymographs, results from KymographClear based analysis and manually annotated ImageJ rois. [file elife-42288-fig2-data2.zip › N30/mov07/frame169.tif]

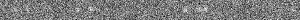

Supplement: Figure 2—source data 2. — A ZIP file containing all analysed synthetic unidirectional movies, their kymographs, results from KymographClear based analysis and manually annotated ImageJ rois. [file elife-42288-fig2-data2.zip › N30/mov07/frame43.tif]

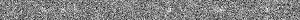

Supplement: Figure 2—source data 2. — A ZIP file containing all analysed synthetic unidirectional movies, their kymographs, results from KymographClear based analysis and manually annotated ImageJ rois. [file elife-42288-fig2-data2.zip › N30/mov07/frame57.tif]

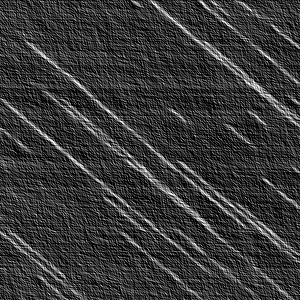

Supplement: Figure 2—source data 2. — A ZIP file containing all analysed synthetic unidirectional movies, their kymographs, results from KymographClear based analysis and manually annotated ImageJ rois. [file elife-42288-fig2-data2.zip › N30/mov07/kymograph/kymograph_1/kymograph_1 filtered_forward.tif]

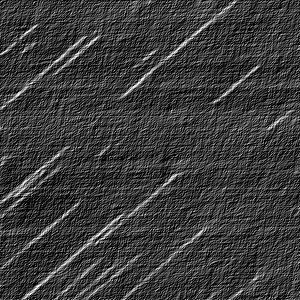

Supplement: Figure 2—source data 2. — A ZIP file containing all analysed synthetic unidirectional movies, their kymographs, results from KymographClear based analysis and manually annotated ImageJ rois. [file elife-42288-fig2-data2.zip › N30/mov07/kymograph/kymograph_1/kymograph_1 filtered_backward.tif]

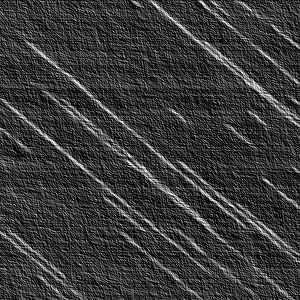

Supplement: Figure 2—source data 2. — A ZIP file containing all analysed synthetic unidirectional movies, their kymographs, results from KymographClear based analysis and manually annotated ImageJ rois. [file elife-42288-fig2-data2.zip › N30/mov07/kymograph/kymograph_1/kymograph_1 color coded directions.tif]

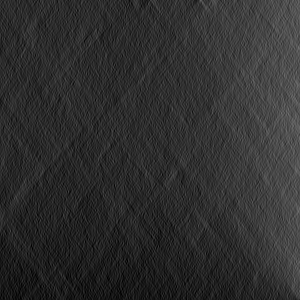

Supplement: Figure 2—source data 2. — A ZIP file containing all analysed synthetic unidirectional movies, their kymographs, results from KymographClear based analysis and manually annotated ImageJ rois. [file elife-42288-fig2-data2.zip › N30/mov07/kymograph/kymograph_1/kymograph_1 static.tif]

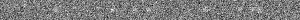

Supplement: Figure 2—source data 2. — A ZIP file containing all analysed synthetic unidirectional movies, their kymographs, results from KymographClear based analysis and manually annotated ImageJ rois. [file elife-42288-fig2-data2.zip › N30/mov07/frame223.tif]

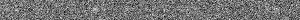

Supplement: Figure 2—source data 2. — A ZIP file containing all analysed synthetic unidirectional movies, their kymographs, results from KymographClear based analysis and manually annotated ImageJ rois. [file elife-42288-fig2-data2.zip › N30/mov07/frame237.tif]

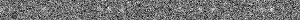

Supplement: Figure 2—source data 2. — A ZIP file containing all analysed synthetic unidirectional movies, their kymographs, results from KymographClear based analysis and manually annotated ImageJ rois. [file elife-42288-fig2-data2.zip › N30/mov07/frame233.tif]

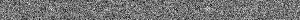

Supplement: Figure 2—source data 2. — A ZIP file containing all analysed synthetic unidirectional movies, their kymographs, results from KymographClear based analysis and manually annotated ImageJ rois. [file elife-42288-fig2-data2.zip › N30/mov07/frame227.tif]

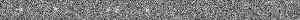

Supplement: Figure 2—source data 2. — A ZIP file containing all analysed synthetic unidirectional movies, their kymographs, results from KymographClear based analysis and manually annotated ImageJ rois. [file elife-42288-fig2-data2.zip › N30/mov07/frame145.tif]

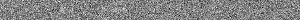

Supplement: Figure 2—source data 2. — A ZIP file containing all analysed synthetic unidirectional movies, their kymographs, results from KymographClear based analysis and manually annotated ImageJ rois. [file elife-42288-fig2-data2.zip › N30/mov07/frame151.tif]

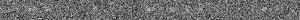

Supplement: Figure 2—source data 2. — A ZIP file containing all analysed synthetic unidirectional movies, their kymographs, results from KymographClear based analysis and manually annotated ImageJ rois. [file elife-42288-fig2-data2.zip › N30/mov07/frame179.tif]

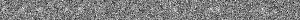

Supplement: Figure 2—source data 2. — A ZIP file containing all analysed synthetic unidirectional movies, their kymographs, results from KymographClear based analysis and manually annotated ImageJ rois. [file elife-42288-fig2-data2.zip › N30/mov07/frame53.tif]

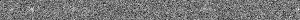

Supplement: Figure 2—source data 2. — A ZIP file containing all analysed synthetic unidirectional movies, their kymographs, results from KymographClear based analysis and manually annotated ImageJ rois. [file elife-42288-fig2-data2.zip › N30/mov07/frame47.tif]

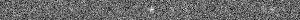

Supplement: Figure 3—source data 2. — A ZIP file containing all analysed synthetic bidirectional movies, their kymographs, and manually annotated ImageJ rois. [file elife-42288-fig3-data2.zip › BN15/mov07/frame242.tif]

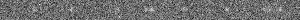

Supplement: Figure 3—source data 2. — A ZIP file containing all analysed synthetic bidirectional movies, their kymographs, and manually annotated ImageJ rois. [file elife-42288-fig3-data2.zip › BN15/mov07/frame256.tif]

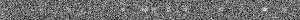

Supplement: Figure 3—source data 2. — A ZIP file containing all analysed synthetic bidirectional movies, their kymographs, and manually annotated ImageJ rois. [file elife-42288-fig3-data2.zip › BN15/mov07/frame281.tif]

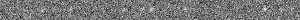

Supplement: Figure 3—source data 2. — A ZIP file containing all analysed synthetic bidirectional movies, their kymographs, and manually annotated ImageJ rois. [file elife-42288-fig3-data2.zip › BN15/mov07/frame295.tif]

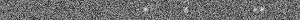

Supplement: Figure 3—source data 2. — A ZIP file containing all analysed synthetic bidirectional movies, their kymographs, and manually annotated ImageJ rois. [file elife-42288-fig3-data2.zip › BN15/mov07/frame108.tif]

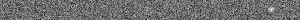

Supplement: Figure 3—source data 2. — A ZIP file containing all analysed synthetic bidirectional movies, their kymographs, and manually annotated ImageJ rois. [file elife-42288-fig3-data2.zip › BN15/mov07/frame22.tif]

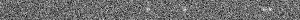

Supplement: Figure 3—source data 2. — A ZIP file containing all analysed synthetic bidirectional movies, their kymographs, and manually annotated ImageJ rois. [file elife-42288-fig3-data2.zip › BN15/mov07/frame36.tif]

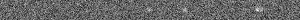

Supplement: Figure 3—source data 2. — A ZIP file containing all analysed synthetic bidirectional movies, their kymographs, and manually annotated ImageJ rois. [file elife-42288-fig3-data2.zip › BN15/mov07/frame134.tif]

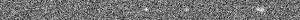

Supplement: Figure 3—source data 2. — A ZIP file containing all analysed synthetic bidirectional movies, their kymographs, and manually annotated ImageJ rois. [file elife-42288-fig3-data2.zip › BN15/mov07/frame120.tif]

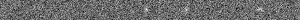

Supplement: Figure 3—source data 2. — A ZIP file containing all analysed synthetic bidirectional movies, their kymographs, and manually annotated ImageJ rois. [file elife-42288-fig3-data2.zip › BN15/mov07/frame121.tif]

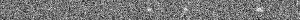

Supplement: Figure 3—source data 2. — A ZIP file containing all analysed synthetic bidirectional movies, their kymographs, and manually annotated ImageJ rois. [file elife-42288-fig3-data2.zip › BN15/mov07/frame135.tif]

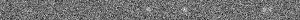

Supplement: Figure 3—source data 2. — A ZIP file containing all analysed synthetic bidirectional movies, their kymographs, and manually annotated ImageJ rois. [file elife-42288-fig3-data2.zip › BN15/mov07/frame37.tif]

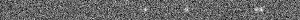

Supplement: Figure 3—source data 2. — A ZIP file containing all analysed synthetic bidirectional movies, their kymographs, and manually annotated ImageJ rois. [file elife-42288-fig3-data2.zip › BN15/mov07/frame109.tif]

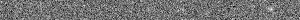

Supplement: Figure 3—source data 2. — A ZIP file containing all analysed synthetic bidirectional movies, their kymographs, and manually annotated ImageJ rois. [file elife-42288-fig3-data2.zip › BN15/mov07/frame23.tif]

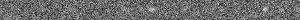

Supplement: Figure 3—source data 2. — A ZIP file containing all analysed synthetic bidirectional movies, their kymographs, and manually annotated ImageJ rois. [file elife-42288-fig3-data2.zip › BN15/mov07/frame294.tif]

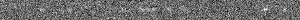

Supplement: Figure 3—source data 2. — A ZIP file containing all analysed synthetic bidirectional movies, their kymographs, and manually annotated ImageJ rois. [file elife-42288-fig3-data2.zip › BN15/mov07/frame280.tif]

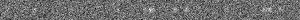

Supplement: Figure 3—source data 2. — A ZIP file containing all analysed synthetic bidirectional movies, their kymographs, and manually annotated ImageJ rois. [file elife-42288-fig3-data2.zip › BN15/mov07/frame257.tif]

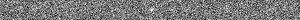

Supplement: Figure 3—source data 2. — A ZIP file containing all analysed synthetic bidirectional movies, their kymographs, and manually annotated ImageJ rois. [file elife-42288-fig3-data2.zip › BN15/mov07/frame243.tif]

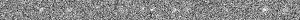

Supplement: Figure 3—source data 2. — A ZIP file containing all analysed synthetic bidirectional movies, their kymographs, and manually annotated ImageJ rois. [file elife-42288-fig3-data2.zip › BN15/mov07/frame269.tif]

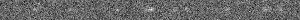

Supplement: Figure 3—source data 2. — A ZIP file containing all analysed synthetic bidirectional movies, their kymographs, and manually annotated ImageJ rois. [file elife-42288-fig3-data2.zip › BN15/mov07/frame255.tif]

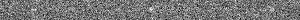

Supplement: Figure 3—source data 2. — A ZIP file containing all analysed synthetic bidirectional movies, their kymographs, and manually annotated ImageJ rois. [file elife-42288-fig3-data2.zip › BN15/mov07/frame241.tif]

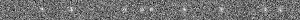

Supplement: Figure 3—source data 2. — A ZIP file containing all analysed synthetic bidirectional movies, their kymographs, and manually annotated ImageJ rois. [file elife-42288-fig3-data2.zip › BN15/mov07/frame296.tif]

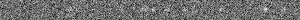

Supplement: Figure 3—source data 2. — A ZIP file containing all analysed synthetic bidirectional movies, their kymographs, and manually annotated ImageJ rois. [file elife-42288-fig3-data2.zip › BN15/mov07/frame282.tif]

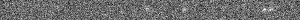

Supplement: Figure 3—source data 2. — A ZIP file containing all analysed synthetic bidirectional movies, their kymographs, and manually annotated ImageJ rois. [file elife-42288-fig3-data2.zip › BN15/mov07/frame35.tif]

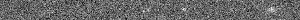

Supplement: Figure 3—source data 2. — A ZIP file containing all analysed synthetic bidirectional movies, their kymographs, and manually annotated ImageJ rois. [file elife-42288-fig3-data2.zip › BN15/mov07/frame21.tif]

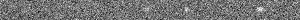

Supplement: Figure 3—source data 2. — A ZIP file containing all analysed synthetic bidirectional movies, their kymographs, and manually annotated ImageJ rois. [file elife-42288-fig3-data2.zip › BN15/mov07/frame123.tif]

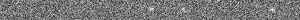

Supplement: Figure 3—source data 2. — A ZIP file containing all analysed synthetic bidirectional movies, their kymographs, and manually annotated ImageJ rois. [file elife-42288-fig3-data2.zip › BN15/mov07/frame8.tif]

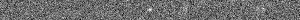

Supplement: Figure 3—source data 2. — A ZIP file containing all analysed synthetic bidirectional movies, their kymographs, and manually annotated ImageJ rois. [file elife-42288-fig3-data2.zip › BN15/mov07/frame137.tif]

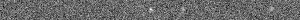

Supplement: Figure 3—source data 2. — A ZIP file containing all analysed synthetic bidirectional movies, their kymographs, and manually annotated ImageJ rois. [file elife-42288-fig3-data2.zip › BN15/mov07/frame9.tif]

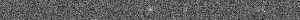

Supplement: Figure 3—source data 2. — A ZIP file containing all analysed synthetic bidirectional movies, their kymographs, and manually annotated ImageJ rois. [file elife-42288-fig3-data2.zip › BN15/mov07/frame136.tif]

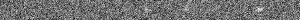

Supplement: Figure 3—source data 2. — A ZIP file containing all analysed synthetic bidirectional movies, their kymographs, and manually annotated ImageJ rois. [file elife-42288-fig3-data2.zip › BN15/mov07/frame122.tif]

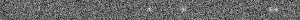

Supplement: Figure 3—source data 2. — A ZIP file containing all analysed synthetic bidirectional movies, their kymographs, and manually annotated ImageJ rois. [file elife-42288-fig3-data2.zip › BN15/mov07/frame20.tif]

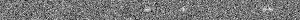

Supplement: Figure 3—source data 2. — A ZIP file containing all analysed synthetic bidirectional movies, their kymographs, and manually annotated ImageJ rois. [file elife-42288-fig3-data2.zip › BN15/mov07/frame34.tif]

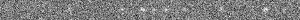

Supplement: Figure 3—source data 2. — A ZIP file containing all analysed synthetic bidirectional movies, their kymographs, and manually annotated ImageJ rois. [file elife-42288-fig3-data2.zip › BN15/mov07/frame283.tif]

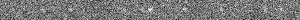

Supplement: Figure 3—source data 2. — A ZIP file containing all analysed synthetic bidirectional movies, their kymographs, and manually annotated ImageJ rois. [file elife-42288-fig3-data2.zip › BN15/mov07/frame297.tif]

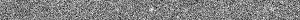

Supplement: Figure 3—source data 2. — A ZIP file containing all analysed synthetic bidirectional movies, their kymographs, and manually annotated ImageJ rois. [file elife-42288-fig3-data2.zip › BN15/mov07/frame240.tif]

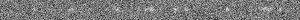

Supplement: Figure 3—source data 2. — A ZIP file containing all analysed synthetic bidirectional movies, their kymographs, and manually annotated ImageJ rois. [file elife-42288-fig3-data2.zip › BN15/mov07/frame254.tif]

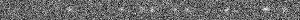

Supplement: Figure 3—source data 2. — A ZIP file containing all analysed synthetic bidirectional movies, their kymographs, and manually annotated ImageJ rois. [file elife-42288-fig3-data2.zip › BN15/mov07/frame268.tif]

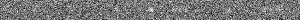

Supplement: Figure 3—source data 2. — A ZIP file containing all analysed synthetic bidirectional movies, their kymographs, and manually annotated ImageJ rois. [file elife-42288-fig3-data2.zip › BN15/mov07/frame250.tif]

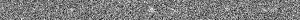

Supplement: Figure 3—source data 2. — A ZIP file containing all analysed synthetic bidirectional movies, their kymographs, and manually annotated ImageJ rois. [file elife-42288-fig3-data2.zip › BN15/mov07/frame244.tif]

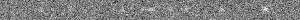

Supplement: Figure 3—source data 2. — A ZIP file containing all analysed synthetic bidirectional movies, their kymographs, and manually annotated ImageJ rois. [file elife-42288-fig3-data2.zip › BN15/mov07/frame278.tif]

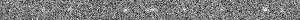

Supplement: Figure 3—source data 2. — A ZIP file containing all analysed synthetic bidirectional movies, their kymographs, and manually annotated ImageJ rois. [file elife-42288-fig3-data2.zip › BN15/mov07/frame293.tif]

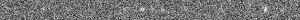

Supplement: Figure 3—source data 2. — A ZIP file containing all analysed synthetic bidirectional movies, their kymographs, and manually annotated ImageJ rois. [file elife-42288-fig3-data2.zip › BN15/mov07/frame287.tif]

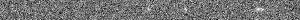

Supplement: Figure 3—source data 2. — A ZIP file containing all analysed synthetic bidirectional movies, their kymographs, and manually annotated ImageJ rois. [file elife-42288-fig3-data2.zip › BN15/mov07/frame126.tif]

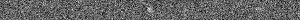

Supplement: Figure 3—source data 2. — A ZIP file containing all analysed synthetic bidirectional movies, their kymographs, and manually annotated ImageJ rois. [file elife-42288-fig3-data2.zip › BN15/mov07/frame132.tif]

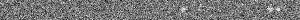

Supplement: Figure 3—source data 2. — A ZIP file containing all analysed synthetic bidirectional movies, their kymographs, and manually annotated ImageJ rois. [file elife-42288-fig3-data2.zip › BN15/mov07/frame18.tif]

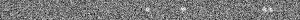

Supplement: Figure 3—source data 2. — A ZIP file containing all analysed synthetic bidirectional movies, their kymographs, and manually annotated ImageJ rois. [file elife-42288-fig3-data2.zip › BN15/mov07/frame30.tif]

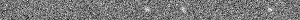

Supplement: Figure 3—source data 2. — A ZIP file containing all analysed synthetic bidirectional movies, their kymographs, and manually annotated ImageJ rois. [file elife-42288-fig3-data2.zip › BN15/mov07/frame24.tif]

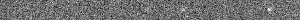

Supplement: Figure 3—source data 2. — A ZIP file containing all analysed synthetic bidirectional movies, their kymographs, and manually annotated ImageJ rois. [file elife-42288-fig3-data2.zip › BN15/mov07/frame25.tif]

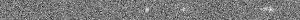

Supplement: Figure 3—source data 2. — A ZIP file containing all analysed synthetic bidirectional movies, their kymographs, and manually annotated ImageJ rois. [file elife-42288-fig3-data2.zip › BN15/mov07/frame31.tif]

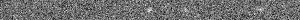

Supplement: Figure 3—source data 2. — A ZIP file containing all analysed synthetic bidirectional movies, their kymographs, and manually annotated ImageJ rois. [file elife-42288-fig3-data2.zip › BN15/mov07/frame133.tif]

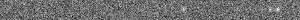

Supplement: Figure 3—source data 2. — A ZIP file containing all analysed synthetic bidirectional movies, their kymographs, and manually annotated ImageJ rois. [file elife-42288-fig3-data2.zip › BN15/mov07/frame19.tif]

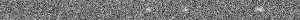

Supplement: Figure 3—source data 2. — A ZIP file containing all analysed synthetic bidirectional movies, their kymographs, and manually annotated ImageJ rois. [file elife-42288-fig3-data2.zip › BN15/mov07/frame127.tif]

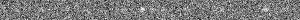

Supplement: Figure 3—source data 2. — A ZIP file containing all analysed synthetic bidirectional movies, their kymographs, and manually annotated ImageJ rois. [file elife-42288-fig3-data2.zip › BN15/mov07/frame286.tif]

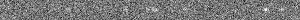

Supplement: Figure 3—source data 2. — A ZIP file containing all analysed synthetic bidirectional movies, their kymographs, and manually annotated ImageJ rois. [file elife-42288-fig3-data2.zip › BN15/mov07/frame292.tif]

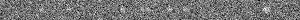

Supplement: Figure 3—source data 2. — A ZIP file containing all analysed synthetic bidirectional movies, their kymographs, and manually annotated ImageJ rois. [file elife-42288-fig3-data2.zip › BN15/mov07/frame279.tif]

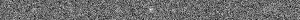

Supplement: Figure 3—source data 2. — A ZIP file containing all analysed synthetic bidirectional movies, their kymographs, and manually annotated ImageJ rois. [file elife-42288-fig3-data2.zip › BN15/mov07/frame245.tif]

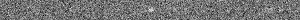

Supplement: Figure 3—source data 2. — A ZIP file containing all analysed synthetic bidirectional movies, their kymographs, and manually annotated ImageJ rois. [file elife-42288-fig3-data2.zip › BN15/mov07/frame251.tif]

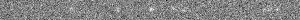

Supplement: Figure 3—source data 2. — A ZIP file containing all analysed synthetic bidirectional movies, their kymographs, and manually annotated ImageJ rois. [file elife-42288-fig3-data2.zip › BN15/mov07/frame247.tif]

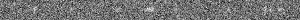

Supplement: Figure 3—source data 2. — A ZIP file containing all analysed synthetic bidirectional movies, their kymographs, and manually annotated ImageJ rois. [file elife-42288-fig3-data2.zip › BN15/mov07/frame253.tif]

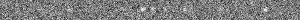

Supplement: Figure 3—source data 2. — A ZIP file containing all analysed synthetic bidirectional movies, their kymographs, and manually annotated ImageJ rois. [file elife-42288-fig3-data2.zip › BN15/mov07/frame284.tif]

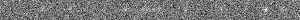

Supplement: Figure 3—source data 2. — A ZIP file containing all analysed synthetic bidirectional movies, their kymographs, and manually annotated ImageJ rois. [file elife-42288-fig3-data2.zip › BN15/mov07/frame290.tif]

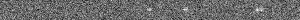

Supplement: Figure 3—source data 2. — A ZIP file containing all analysed synthetic bidirectional movies, their kymographs, and manually annotated ImageJ rois. [file elife-42288-fig3-data2.zip › BN15/mov07/frame131.tif]

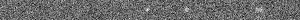

Supplement: Figure 3—source data 2. — A ZIP file containing all analysed synthetic bidirectional movies, their kymographs, and manually annotated ImageJ rois. [file elife-42288-fig3-data2.zip › BN15/mov07/frame125.tif]

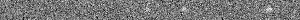

Supplement: Figure 3—source data 2. — A ZIP file containing all analysed synthetic bidirectional movies, their kymographs, and manually annotated ImageJ rois. [file elife-42288-fig3-data2.zip › BN15/mov07/frame27.tif]

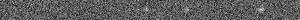

Supplement: Figure 3—source data 2. — A ZIP file containing all analysed synthetic bidirectional movies, their kymographs, and manually annotated ImageJ rois. [file elife-42288-fig3-data2.zip › BN15/mov07/frame119.tif]

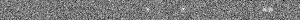

Supplement: Figure 3—source data 2. — A ZIP file containing all analysed synthetic bidirectional movies, their kymographs, and manually annotated ImageJ rois. [file elife-42288-fig3-data2.zip › BN15/mov07/frame33.tif]
