# Supplementary figures and images for: KymoButler, a deep learning software for automated kymograph analysis (part 4 of 4)
Source: eLife. 2019 Aug 13;8:e42288. doi: 10.7554/eLife.42288 (PMC6692109; doi:10.7554/eLife.42288)

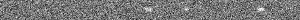

Supplement: Figure 3—source data 2. — A ZIP file containing all analysed synthetic bidirectional movies, their kymographs, and manually annotated ImageJ rois. [file elife-42288-fig3-data2.zip › BN15/mov07/frame153.tif]

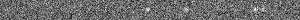

Supplement: Figure 3—source data 2. — A ZIP file containing all analysed synthetic bidirectional movies, their kymographs, and manually annotated ImageJ rois. [file elife-42288-fig3-data2.zip › BN15/mov07/frame79.tif]

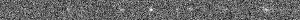

Supplement: Figure 3—source data 2. — A ZIP file containing all analysed synthetic bidirectional movies, their kymographs, and manually annotated ImageJ rois. [file elife-42288-fig3-data2.zip › BN15/mov07/frame219.tif]

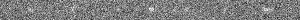

Supplement: Figure 3—source data 2. — A ZIP file containing all analysed synthetic bidirectional movies, their kymographs, and manually annotated ImageJ rois. [file elife-42288-fig3-data2.zip › BN15/mov07/frame231.tif]

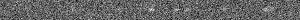

Supplement: Figure 3—source data 2. — A ZIP file containing all analysed synthetic bidirectional movies, their kymographs, and manually annotated ImageJ rois. [file elife-42288-fig3-data2.zip › BN15/mov07/frame225.tif]

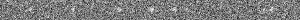

Supplement: Figure 3—source data 2. — A ZIP file containing all analysed synthetic bidirectional movies, their kymographs, and manually annotated ImageJ rois. [file elife-42288-fig3-data2.zip › BN15/mov07/frame228.tif]

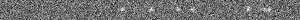

Supplement: Figure 3—source data 2. — A ZIP file containing all analysed synthetic bidirectional movies, their kymographs, and manually annotated ImageJ rois. [file elife-42288-fig3-data2.zip › BN15/mov07/frame200.tif]

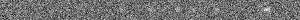

Supplement: Figure 3—source data 2. — A ZIP file containing all analysed synthetic bidirectional movies, their kymographs, and manually annotated ImageJ rois. [file elife-42288-fig3-data2.zip › BN15/mov07/frame214.tif]
