# Supplementary figures and images for: KymoButler, a deep learning software for automated kymograph analysis (part 3 of 4)
Source: eLife. 2019 Aug 13;8:e42288. doi: 10.7554/eLife.42288 (PMC6692109; doi:10.7554/eLife.42288)

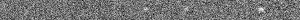

Supplement: Figure 3—source data 2. — A ZIP file containing all analysed synthetic bidirectional movies, their kymographs, and manually annotated ImageJ rois. [file elife-42288-fig3-data2.zip › BN15/mov07/frame118.tif]

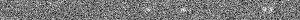

Supplement: Figure 3—source data 2. — A ZIP file containing all analysed synthetic bidirectional movies, their kymographs, and manually annotated ImageJ rois. [file elife-42288-fig3-data2.zip › BN15/mov07/frame32.tif]

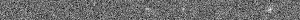

Supplement: Figure 3—source data 2. — A ZIP file containing all analysed synthetic bidirectional movies, their kymographs, and manually annotated ImageJ rois. [file elife-42288-fig3-data2.zip › BN15/mov07/frame26.tif]

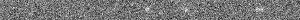

Supplement: Figure 3—source data 2. — A ZIP file containing all analysed synthetic bidirectional movies, their kymographs, and manually annotated ImageJ rois. [file elife-42288-fig3-data2.zip › BN15/mov07/frame124.tif]

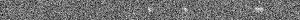

Supplement: Figure 3—source data 2. — A ZIP file containing all analysed synthetic bidirectional movies, their kymographs, and manually annotated ImageJ rois. [file elife-42288-fig3-data2.zip › BN15/mov07/frame130.tif]

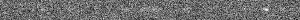

Supplement: Figure 3—source data 2. — A ZIP file containing all analysed synthetic bidirectional movies, their kymographs, and manually annotated ImageJ rois. [file elife-42288-fig3-data2.zip › BN15/mov07/frame291.tif]

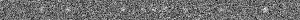

Supplement: Figure 3—source data 2. — A ZIP file containing all analysed synthetic bidirectional movies, their kymographs, and manually annotated ImageJ rois. [file elife-42288-fig3-data2.zip › BN15/mov07/frame285.tif]

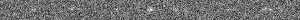

Supplement: Figure 3—source data 2. — A ZIP file containing all analysed synthetic bidirectional movies, their kymographs, and manually annotated ImageJ rois. [file elife-42288-fig3-data2.zip › BN15/mov07/frame252.tif]

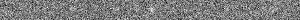

Supplement: Figure 3—source data 2. — A ZIP file containing all analysed synthetic bidirectional movies, their kymographs, and manually annotated ImageJ rois. [file elife-42288-fig3-data2.zip › BN15/mov07/frame246.tif]

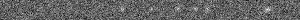

Supplement: Figure 3—source data 2. — A ZIP file containing all analysed synthetic bidirectional movies, their kymographs, and manually annotated ImageJ rois. [file elife-42288-fig3-data2.zip › BN15/mov07/frame209.tif]

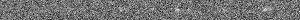

Supplement: Figure 3—source data 2. — A ZIP file containing all analysed synthetic bidirectional movies, their kymographs, and manually annotated ImageJ rois. [file elife-42288-fig3-data2.zip › BN15/mov07/frame221.tif]

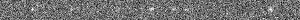

Supplement: Figure 3—source data 2. — A ZIP file containing all analysed synthetic bidirectional movies, their kymographs, and manually annotated ImageJ rois. [file elife-42288-fig3-data2.zip › BN15/mov07/frame235.tif]

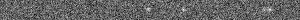

Supplement: Figure 3—source data 2. — A ZIP file containing all analysed synthetic bidirectional movies, their kymographs, and manually annotated ImageJ rois. [file elife-42288-fig3-data2.zip › BN15/mov07/frame41.tif]

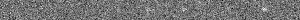

Supplement: Figure 3—source data 2. — A ZIP file containing all analysed synthetic bidirectional movies, their kymographs, and manually annotated ImageJ rois. [file elife-42288-fig3-data2.zip › BN15/mov07/frame55.tif]

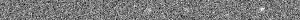

Supplement: Figure 3—source data 2. — A ZIP file containing all analysed synthetic bidirectional movies, their kymographs, and manually annotated ImageJ rois. [file elife-42288-fig3-data2.zip › BN15/mov07/frame157.tif]

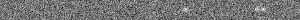

Supplement: Figure 3—source data 2. — A ZIP file containing all analysed synthetic bidirectional movies, their kymographs, and manually annotated ImageJ rois. [file elife-42288-fig3-data2.zip › BN15/mov07/frame143.tif]

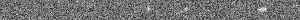

Supplement: Figure 3—source data 2. — A ZIP file containing all analysed synthetic bidirectional movies, their kymographs, and manually annotated ImageJ rois. [file elife-42288-fig3-data2.zip › BN15/mov07/frame69.tif]

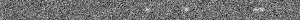

Supplement: Figure 3—source data 2. — A ZIP file containing all analysed synthetic bidirectional movies, their kymographs, and manually annotated ImageJ rois. [file elife-42288-fig3-data2.zip › BN15/mov07/frame82.tif]

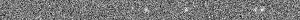

Supplement: Figure 3—source data 2. — A ZIP file containing all analysed synthetic bidirectional movies, their kymographs, and manually annotated ImageJ rois. [file elife-42288-fig3-data2.zip › BN15/mov07/frame96.tif]

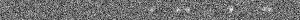

Supplement: Figure 3—source data 2. — A ZIP file containing all analysed synthetic bidirectional movies, their kymographs, and manually annotated ImageJ rois. [file elife-42288-fig3-data2.zip › BN15/mov07/frame194.tif]

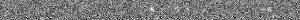

Supplement: Figure 3—source data 2. — A ZIP file containing all analysed synthetic bidirectional movies, their kymographs, and manually annotated ImageJ rois. [file elife-42288-fig3-data2.zip › BN15/mov07/frame180.tif]

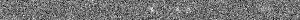

Supplement: Figure 3—source data 2. — A ZIP file containing all analysed synthetic bidirectional movies, their kymographs, and manually annotated ImageJ rois. [file elife-42288-fig3-data2.zip › BN15/mov07/frame181.tif]

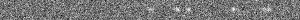

Supplement: Figure 3—source data 2. — A ZIP file containing all analysed synthetic bidirectional movies, their kymographs, and manually annotated ImageJ rois. [file elife-42288-fig3-data2.zip › BN15/mov07/frame195.tif]

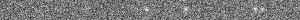

Supplement: Figure 3—source data 2. — A ZIP file containing all analysed synthetic bidirectional movies, their kymographs, and manually annotated ImageJ rois. [file elife-42288-fig3-data2.zip › BN15/mov07/frame97.tif]

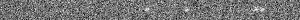

Supplement: Figure 3—source data 2. — A ZIP file containing all analysed synthetic bidirectional movies, their kymographs, and manually annotated ImageJ rois. [file elife-42288-fig3-data2.zip › BN15/mov07/frame83.tif]

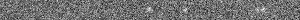

Supplement: Figure 3—source data 2. — A ZIP file containing all analysed synthetic bidirectional movies, their kymographs, and manually annotated ImageJ rois. [file elife-42288-fig3-data2.zip › BN15/mov07/frame142.tif]

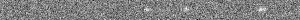

Supplement: Figure 3—source data 2. — A ZIP file containing all analysed synthetic bidirectional movies, their kymographs, and manually annotated ImageJ rois. [file elife-42288-fig3-data2.zip › BN15/mov07/frame68.tif]

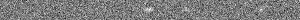

Supplement: Figure 3—source data 2. — A ZIP file containing all analysed synthetic bidirectional movies, their kymographs, and manually annotated ImageJ rois. [file elife-42288-fig3-data2.zip › BN15/mov07/frame156.tif]

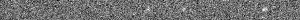

Supplement: Figure 3—source data 2. — A ZIP file containing all analysed synthetic bidirectional movies, their kymographs, and manually annotated ImageJ rois. [file elife-42288-fig3-data2.zip › BN15/mov07/frame54.tif]

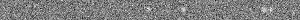

Supplement: Figure 3—source data 2. — A ZIP file containing all analysed synthetic bidirectional movies, their kymographs, and manually annotated ImageJ rois. [file elife-42288-fig3-data2.zip › BN15/mov07/frame40.tif]

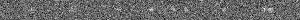

Supplement: Figure 3—source data 2. — A ZIP file containing all analysed synthetic bidirectional movies, their kymographs, and manually annotated ImageJ rois. [file elife-42288-fig3-data2.zip › BN15/mov07/frame234.tif]

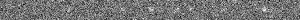

Supplement: Figure 3—source data 2. — A ZIP file containing all analysed synthetic bidirectional movies, their kymographs, and manually annotated ImageJ rois. [file elife-42288-fig3-data2.zip › BN15/mov07/frame220.tif]

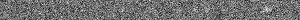

Supplement: Figure 3—source data 2. — A ZIP file containing all analysed synthetic bidirectional movies, their kymographs, and manually annotated ImageJ rois. [file elife-42288-fig3-data2.zip › BN15/mov07/frame208.tif]

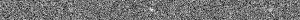

Supplement: Figure 3—source data 2. — A ZIP file containing all analysed synthetic bidirectional movies, their kymographs, and manually annotated ImageJ rois. [file elife-42288-fig3-data2.zip › BN15/mov07/frame236.tif]

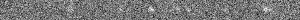

Supplement: Figure 3—source data 2. — A ZIP file containing all analysed synthetic bidirectional movies, their kymographs, and manually annotated ImageJ rois. [file elife-42288-fig3-data2.zip › BN15/mov07/frame222.tif]

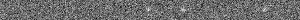

Supplement: Figure 3—source data 2. — A ZIP file containing all analysed synthetic bidirectional movies, their kymographs, and manually annotated ImageJ rois. [file elife-42288-fig3-data2.zip › BN15/mov07/frame56.tif]

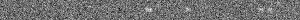

Supplement: Figure 3—source data 2. — A ZIP file containing all analysed synthetic bidirectional movies, their kymographs, and manually annotated ImageJ rois. [file elife-42288-fig3-data2.zip › BN15/mov07/frame168.tif]

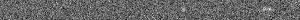

Supplement: Figure 3—source data 2. — A ZIP file containing all analysed synthetic bidirectional movies, their kymographs, and manually annotated ImageJ rois. [file elife-42288-fig3-data2.zip › BN15/mov07/frame42.tif]

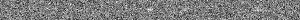

Supplement: Figure 3—source data 2. — A ZIP file containing all analysed synthetic bidirectional movies, their kymographs, and manually annotated ImageJ rois. [file elife-42288-fig3-data2.zip › BN15/mov07/frame140.tif]

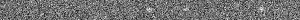

Supplement: Figure 3—source data 2. — A ZIP file containing all analysed synthetic bidirectional movies, their kymographs, and manually annotated ImageJ rois. [file elife-42288-fig3-data2.zip › BN15/mov07/frame154.tif]

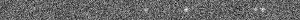

Supplement: Figure 3—source data 2. — A ZIP file containing all analysed synthetic bidirectional movies, their kymographs, and manually annotated ImageJ rois. [file elife-42288-fig3-data2.zip › BN15/mov07/frame95.tif]

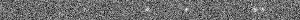

Supplement: Figure 3—source data 2. — A ZIP file containing all analysed synthetic bidirectional movies, their kymographs, and manually annotated ImageJ rois. [file elife-42288-fig3-data2.zip › BN15/mov07/frame81.tif]

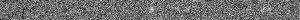

Supplement: Figure 3—source data 2. — A ZIP file containing all analysed synthetic bidirectional movies, their kymographs, and manually annotated ImageJ rois. [file elife-42288-fig3-data2.zip › BN15/mov07/frame183.tif]

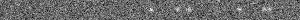

Supplement: Figure 3—source data 2. — A ZIP file containing all analysed synthetic bidirectional movies, their kymographs, and manually annotated ImageJ rois. [file elife-42288-fig3-data2.zip › BN15/mov07/frame197.tif]

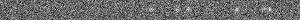

Supplement: Figure 3—source data 2. — A ZIP file containing all analysed synthetic bidirectional movies, their kymographs, and manually annotated ImageJ rois. [file elife-42288-fig3-data2.zip › BN15/mov07/frame196.tif]

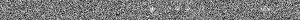

Supplement: Figure 3—source data 2. — A ZIP file containing all analysed synthetic bidirectional movies, their kymographs, and manually annotated ImageJ rois. [file elife-42288-fig3-data2.zip › BN15/mov07/frame182.tif]

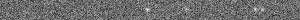

Supplement: Figure 3—source data 2. — A ZIP file containing all analysed synthetic bidirectional movies, their kymographs, and manually annotated ImageJ rois. [file elife-42288-fig3-data2.zip › BN15/mov07/frame80.tif]

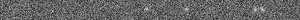

Supplement: Figure 3—source data 2. — A ZIP file containing all analysed synthetic bidirectional movies, their kymographs, and manually annotated ImageJ rois. [file elife-42288-fig3-data2.zip › BN15/mov07/frame94.tif]

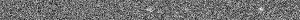

Supplement: Figure 3—source data 2. — A ZIP file containing all analysed synthetic bidirectional movies, their kymographs, and manually annotated ImageJ rois. [file elife-42288-fig3-data2.zip › BN15/mov07/frame155.tif]

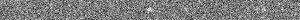

Supplement: Figure 3—source data 2. — A ZIP file containing all analysed synthetic bidirectional movies, their kymographs, and manually annotated ImageJ rois. [file elife-42288-fig3-data2.zip › BN15/mov07/frame141.tif]

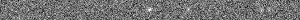

Supplement: Figure 3—source data 2. — A ZIP file containing all analysed synthetic bidirectional movies, their kymographs, and manually annotated ImageJ rois. [file elife-42288-fig3-data2.zip › BN15/mov07/frame169.tif]

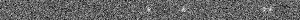

Supplement: Figure 3—source data 2. — A ZIP file containing all analysed synthetic bidirectional movies, their kymographs, and manually annotated ImageJ rois. [file elife-42288-fig3-data2.zip › BN15/mov07/frame43.tif]

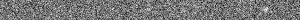

Supplement: Figure 3—source data 2. — A ZIP file containing all analysed synthetic bidirectional movies, their kymographs, and manually annotated ImageJ rois. [file elife-42288-fig3-data2.zip › BN15/mov07/frame57.tif]

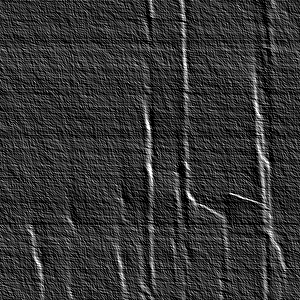

Supplement: Figure 3—source data 2. — A ZIP file containing all analysed synthetic bidirectional movies, their kymographs, and manually annotated ImageJ rois. [file elife-42288-fig3-data2.zip › BN15/mov07/kymograph/kymograph_1/kymograph_1 filtered_forward.tif]

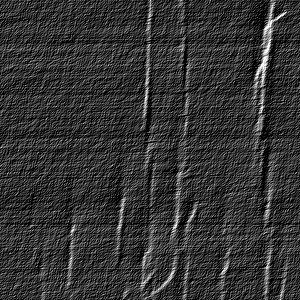

Supplement: Figure 3—source data 2. — A ZIP file containing all analysed synthetic bidirectional movies, their kymographs, and manually annotated ImageJ rois. [file elife-42288-fig3-data2.zip › BN15/mov07/kymograph/kymograph_1/kymograph_1 filtered_backward.tif]

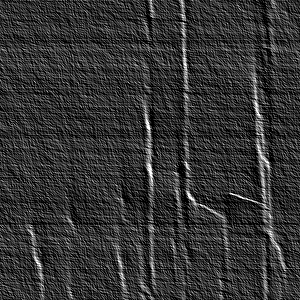

Supplement: Figure 3—source data 2. — A ZIP file containing all analysed synthetic bidirectional movies, their kymographs, and manually annotated ImageJ rois. [file elife-42288-fig3-data2.zip › BN15/mov07/kymograph/kymograph_1/kymograph_1 color coded directions.tif]

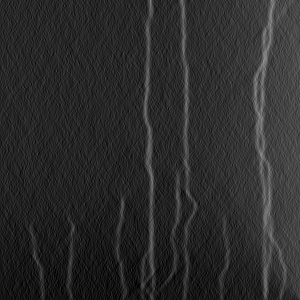

Supplement: Figure 3—source data 2. — A ZIP file containing all analysed synthetic bidirectional movies, their kymographs, and manually annotated ImageJ rois. [file elife-42288-fig3-data2.zip › BN15/mov07/kymograph/kymograph_1/kymograph_1 static.tif]

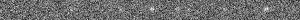

Supplement: Figure 3—source data 2. — A ZIP file containing all analysed synthetic bidirectional movies, their kymographs, and manually annotated ImageJ rois. [file elife-42288-fig3-data2.zip › BN15/mov07/frame223.tif]

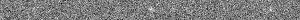

Supplement: Figure 3—source data 2. — A ZIP file containing all analysed synthetic bidirectional movies, their kymographs, and manually annotated ImageJ rois. [file elife-42288-fig3-data2.zip › BN15/mov07/frame237.tif]

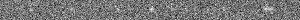

Supplement: Figure 3—source data 2. — A ZIP file containing all analysed synthetic bidirectional movies, their kymographs, and manually annotated ImageJ rois. [file elife-42288-fig3-data2.zip › BN15/mov07/frame233.tif]

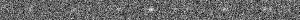

Supplement: Figure 3—source data 2. — A ZIP file containing all analysed synthetic bidirectional movies, their kymographs, and manually annotated ImageJ rois. [file elife-42288-fig3-data2.zip › BN15/mov07/frame227.tif]

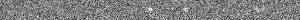

Supplement: Figure 3—source data 2. — A ZIP file containing all analysed synthetic bidirectional movies, their kymographs, and manually annotated ImageJ rois. [file elife-42288-fig3-data2.zip › BN15/mov07/frame145.tif]

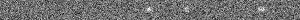

Supplement: Figure 3—source data 2. — A ZIP file containing all analysed synthetic bidirectional movies, their kymographs, and manually annotated ImageJ rois. [file elife-42288-fig3-data2.zip › BN15/mov07/frame151.tif]

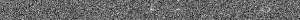

Supplement: Figure 3—source data 2. — A ZIP file containing all analysed synthetic bidirectional movies, their kymographs, and manually annotated ImageJ rois. [file elife-42288-fig3-data2.zip › BN15/mov07/frame179.tif]

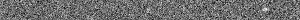

Supplement: Figure 3—source data 2. — A ZIP file containing all analysed synthetic bidirectional movies, their kymographs, and manually annotated ImageJ rois. [file elife-42288-fig3-data2.zip › BN15/mov07/frame53.tif]

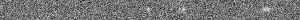

Supplement: Figure 3—source data 2. — A ZIP file containing all analysed synthetic bidirectional movies, their kymographs, and manually annotated ImageJ rois. [file elife-42288-fig3-data2.zip › BN15/mov07/frame47.tif]

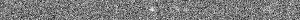

Supplement: Figure 3—source data 2. — A ZIP file containing all analysed synthetic bidirectional movies, their kymographs, and manually annotated ImageJ rois. [file elife-42288-fig3-data2.zip › BN15/mov07/frame186.tif]

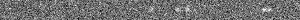

Supplement: Figure 3—source data 2. — A ZIP file containing all analysed synthetic bidirectional movies, their kymographs, and manually annotated ImageJ rois. [file elife-42288-fig3-data2.zip › BN15/mov07/frame192.tif]

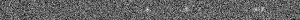

Supplement: Figure 3—source data 2. — A ZIP file containing all analysed synthetic bidirectional movies, their kymographs, and manually annotated ImageJ rois. [file elife-42288-fig3-data2.zip › BN15/mov07/frame90.tif]

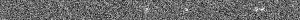

Supplement: Figure 3—source data 2. — A ZIP file containing all analysed synthetic bidirectional movies, their kymographs, and manually annotated ImageJ rois. [file elife-42288-fig3-data2.zip › BN15/mov07/frame84.tif]

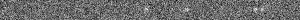

Supplement: Figure 3—source data 2. — A ZIP file containing all analysed synthetic bidirectional movies, their kymographs, and manually annotated ImageJ rois. [file elife-42288-fig3-data2.zip › BN15/mov07/frame85.tif]

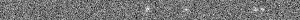

Supplement: Figure 3—source data 2. — A ZIP file containing all analysed synthetic bidirectional movies, their kymographs, and manually annotated ImageJ rois. [file elife-42288-fig3-data2.zip › BN15/mov07/frame91.tif]

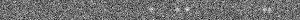

Supplement: Figure 3—source data 2. — A ZIP file containing all analysed synthetic bidirectional movies, their kymographs, and manually annotated ImageJ rois. [file elife-42288-fig3-data2.zip › BN15/mov07/frame193.tif]

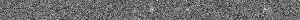

Supplement: Figure 3—source data 2. — A ZIP file containing all analysed synthetic bidirectional movies, their kymographs, and manually annotated ImageJ rois. [file elife-42288-fig3-data2.zip › BN15/mov07/frame187.tif]

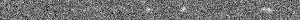

Supplement: Figure 3—source data 2. — A ZIP file containing all analysed synthetic bidirectional movies, their kymographs, and manually annotated ImageJ rois. [file elife-42288-fig3-data2.zip › BN15/mov07/frame46.tif]

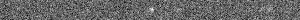

Supplement: Figure 3—source data 2. — A ZIP file containing all analysed synthetic bidirectional movies, their kymographs, and manually annotated ImageJ rois. [file elife-42288-fig3-data2.zip › BN15/mov07/frame178.tif]

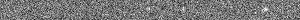

Supplement: Figure 3—source data 2. — A ZIP file containing all analysed synthetic bidirectional movies, their kymographs, and manually annotated ImageJ rois. [file elife-42288-fig3-data2.zip › BN15/mov07/frame52.tif]

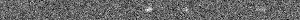

Supplement: Figure 3—source data 2. — A ZIP file containing all analysed synthetic bidirectional movies, their kymographs, and manually annotated ImageJ rois. [file elife-42288-fig3-data2.zip › BN15/mov07/frame150.tif]

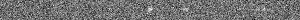

Supplement: Figure 3—source data 2. — A ZIP file containing all analysed synthetic bidirectional movies, their kymographs, and manually annotated ImageJ rois. [file elife-42288-fig3-data2.zip › BN15/mov07/frame144.tif]

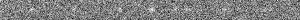

Supplement: Figure 3—source data 2. — A ZIP file containing all analysed synthetic bidirectional movies, their kymographs, and manually annotated ImageJ rois. [file elife-42288-fig3-data2.zip › BN15/mov07/frame226.tif]

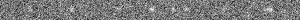

Supplement: Figure 3—source data 2. — A ZIP file containing all analysed synthetic bidirectional movies, their kymographs, and manually annotated ImageJ rois. [file elife-42288-fig3-data2.zip › BN15/mov07/frame232.tif]

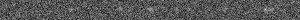

Supplement: Figure 3—source data 2. — A ZIP file containing all analysed synthetic bidirectional movies, their kymographs, and manually annotated ImageJ rois. [file elife-42288-fig3-data2.zip › BN15/mov07/frame224.tif]

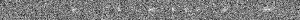

Supplement: Figure 3—source data 2. — A ZIP file containing all analysed synthetic bidirectional movies, their kymographs, and manually annotated ImageJ rois. [file elife-42288-fig3-data2.zip › BN15/mov07/frame230.tif]

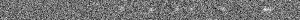

Supplement: Figure 3—source data 2. — A ZIP file containing all analysed synthetic bidirectional movies, their kymographs, and manually annotated ImageJ rois. [file elife-42288-fig3-data2.zip › BN15/mov07/frame218.tif]

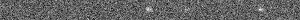

Supplement: Figure 3—source data 2. — A ZIP file containing all analysed synthetic bidirectional movies, their kymographs, and manually annotated ImageJ rois. [file elife-42288-fig3-data2.zip › BN15/mov07/frame152.tif]

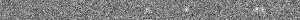

Supplement: Figure 3—source data 2. — A ZIP file containing all analysed synthetic bidirectional movies, their kymographs, and manually annotated ImageJ rois. [file elife-42288-fig3-data2.zip › BN15/mov07/frame78.tif]

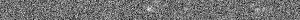

Supplement: Figure 3—source data 2. — A ZIP file containing all analysed synthetic bidirectional movies, their kymographs, and manually annotated ImageJ rois. [file elife-42288-fig3-data2.zip › BN15/mov07/frame146.tif]

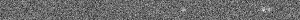

Supplement: Figure 3—source data 2. — A ZIP file containing all analysed synthetic bidirectional movies, their kymographs, and manually annotated ImageJ rois. [file elife-42288-fig3-data2.zip › BN15/mov07/frame44.tif]

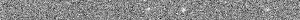

Supplement: Figure 3—source data 2. — A ZIP file containing all analysed synthetic bidirectional movies, their kymographs, and manually annotated ImageJ rois. [file elife-42288-fig3-data2.zip › BN15/mov07/frame50.tif]

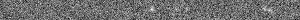

Supplement: Figure 3—source data 2. — A ZIP file containing all analysed synthetic bidirectional movies, their kymographs, and manually annotated ImageJ rois. [file elife-42288-fig3-data2.zip › BN15/mov07/frame191.tif]

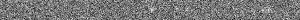

Supplement: Figure 3—source data 2. — A ZIP file containing all analysed synthetic bidirectional movies, their kymographs, and manually annotated ImageJ rois. [file elife-42288-fig3-data2.zip › BN15/mov07/frame185.tif]

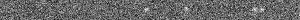

Supplement: Figure 3—source data 2. — A ZIP file containing all analysed synthetic bidirectional movies, their kymographs, and manually annotated ImageJ rois. [file elife-42288-fig3-data2.zip › BN15/mov07/frame87.tif]

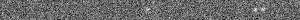

Supplement: Figure 3—source data 2. — A ZIP file containing all analysed synthetic bidirectional movies, their kymographs, and manually annotated ImageJ rois. [file elife-42288-fig3-data2.zip › BN15/mov07/frame93.tif]

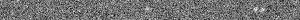

Supplement: Figure 3—source data 2. — A ZIP file containing all analysed synthetic bidirectional movies, their kymographs, and manually annotated ImageJ rois. [file elife-42288-fig3-data2.zip › BN15/mov07/frame92.tif]

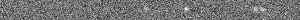

Supplement: Figure 3—source data 2. — A ZIP file containing all analysed synthetic bidirectional movies, their kymographs, and manually annotated ImageJ rois. [file elife-42288-fig3-data2.zip › BN15/mov07/frame86.tif]

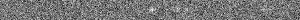

Supplement: Figure 3—source data 2. — A ZIP file containing all analysed synthetic bidirectional movies, their kymographs, and manually annotated ImageJ rois. [file elife-42288-fig3-data2.zip › BN15/mov07/frame184.tif]

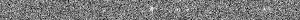

Supplement: Figure 3—source data 2. — A ZIP file containing all analysed synthetic bidirectional movies, their kymographs, and manually annotated ImageJ rois. [file elife-42288-fig3-data2.zip › BN15/mov07/frame190.tif]

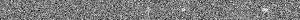

Supplement: Figure 3—source data 2. — A ZIP file containing all analysed synthetic bidirectional movies, their kymographs, and manually annotated ImageJ rois. [file elife-42288-fig3-data2.zip › BN15/mov07/frame51.tif]

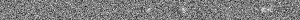

Supplement: Figure 3—source data 2. — A ZIP file containing all analysed synthetic bidirectional movies, their kymographs, and manually annotated ImageJ rois. [file elife-42288-fig3-data2.zip › BN15/mov07/frame45.tif]

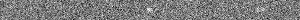

Supplement: Figure 3—source data 2. — A ZIP file containing all analysed synthetic bidirectional movies, their kymographs, and manually annotated ImageJ rois. [file elife-42288-fig3-data2.zip › BN15/mov07/frame147.tif]
